# Supplementary material for: Evaluating implementation of LEAPS, a youth-led early childhood care and education intervention in rural Pakistan: protocol for a stepped wedge cluster-randomized trial
Source: Trials. 2021 Aug 17;22:542. doi: 10.1186/s13063-021-05518-9 (PMC8371849; doi:10.1186/s13063-021-05518-9)
Supplement: Supplementary file 1 — Additional file 1. Supplemental tables and figures. [file 13063_2021_5518_MOESM1_ESM.docx]

**Additional file 1. Supplemental tables and figures**

**Table 1.** Child and parental education in rural Sindh, 2019. Summary of school enrollment, completion, and learning outcomes for children and their caregivers in rural communities of Sindh Province, Pakistan (ASER Pakistan, 2020).

| **Indicator** | **Outcomes in rural Sindh** |
| --- | --- |
| ***Preschool Enrollment (aged 3-5 years)*** | |
| Overall | 45.6% |
| By Age Group |  |
| 3 years | 21.0% |
| 4 years | 67.2% |
| 5 years | 23.4% |
| ***Basic Education Enrollment (aged 6-16 years)*** | |
| Overall | 86.2% |
| By Age Group |  |
| 6-10 years | 84.7% |
| 11-13 years | 92.2% |
| 14-16 years | 84.6% |
| By Gender |  |
| Boys | 62.4% |
| Girls | 37.6% |
| ***Child Learning Outcomes (aged 5-16 years)*** | |
| *Learning Levels, Sindhi (5-16 years)* |  |
| Grade 1: Can read letters | 44.4% |
| Grade 3: Can read sentences | 23.5% |
| Grade 5: Can read stories | 24.0% |
| Can read at least sentences: |  |
| Boys | 33.0% |
| Girls | 25.0% |
| *Learning Levels, English (6-16 years)* |  |
| Grade 1: Can read letters | 38.8% |
| Grade 3: Can read words | 26.9% |
| Grade 5: Can read sentences | 26.6% |
| Can read at least words: |  |
| Boys | 32.0% |
| Girls | 24.0% |
| *Learning Levels, Arithmetic (6-16 years)* |  |
| Grade 1: Can recognize numbers 1-99 | 48.2% |
| Grade 3: Can do two-digit subtraction | 21.7% |
| Grade 5: Can do two-digit division | 30.5% |
| Can at least do subtraction: |  |
| Boys | 28% |
| Girls | 22% |
| ***Parental Education (among parents with children aged 6-16 years)*** | |
| Completed Primary School: |  |
| Mothers | 26% |
| Fathers | 44% |

**Table 2.** Youth education & employment in rural Sindh and rural Pakistan, 2018-2019. Summary of literacy rates, school attendance and school completion among male and female youth aged 15-24 years in rural Sindh, and school completion and national workforce participation rates among male and female youth aged 15-24 years in rural communities (Pakistan Bureau of Statistics, 2018; Pakistan Bureau of Statistics, 2020).

|  | **Male** | | **Female** | |
| --- | --- | --- | --- | --- |
|  | **15-19 years** | **20-24 years** | **15-19 years** | **20-24 years** |
| ***Rural Sindh*** | | | | |
| Youth Literacy | 61% | | 30% | |
| Ever Attended School | 76.1% | 64.0% | 76.4% | 59.4% |
| Top Reasons for Leaving School Early | Student not willing (50%) | | Student not willing (30%) | |
|  | Had to help at work (10%) | | Parents didn’t allow (16%) | |
|  | Other (27%) | | Other (24%) | |
| ***Rural Pakistan*** | | | | |
| Completed Primary School | 72.0% | 69.0% | 54% | 48% |
| Labor Force Participation Rate | 47.6% | 84.6% | 15.6% | 23.3% |
| Unemployment Rate | 11.6% | 10.5% | 6.5% | 15.2% |

**Table 3.** Summary of LEAPS intervention delivery. This table outlines the intervention dosage, location, modes of delivery, and tools for monitoring and evaluation for the LEAPS program. CYL= Community Youth Leader; FO= Field Officer; LIST = LEAPS Intervention Support TEAM; NCHD = National Commission for Human Development.

| **Type of Activity** | **No. of days** | **Duration/day in hours** | **Location** | **Mode of delivery** | **Tools for monitoring & evaluation and quality improvement** |
| --- | --- | --- | --- | --- | --- |
| *Training of Trainers* | 5 days | 8 hours/day | Community event space | In-person,  Group | Pre- and post-test |
| *Coaching for Master Trainers by LIST members* | 1 prep day, plus coaching throughout CYL training days | 8-hour prep day, plus coaching during CYL training | NCHD Office, community event space | In-person, Group and  Individual support | Checklist tool |
| *CYL training* | 12 days | 8 hours/day | NCHD Office, community event space | In-person,  Group | Pre- and post-test |
| *Refresher trainings for CYLs and FOs* | 1 day/training | 8 hours/day | NCHD Office, community event space | In-person,  Group | Pre- and post-test |
| *Supervision of FOs by LIST members* | One visit/month, plus follow-ups by phone | 3 hours/visit | LEAPS preschool | In-person and virtual,  Individual support | Checklist tool |
| *Supervision of CYLs by FOs* | Once/month | 3 hours/visit | LEAPS preschool | In-person,  Individual support | Checklist tool |
| *LEAPS preschool* | 6 days/week (Monday to Saturday) | 3 hours/ session | LEAPS preschool | In-person,  Group | Attendance registers and child progress forms |

**Table 4.** Overview of LEAPS CYL Training. Summarizes the 12-day CYL training in detail, including the duration, format, training content, and manuals and forms given to CYLs; CYL=Community Youth Leader; NCHD = National Commission for Human Development; LIST = LEAPS Intervention Support Team.

| **Duration and Format of Training** | **Training Content** | **Manuals and forms given to CYLs** |
| --- | --- | --- |
| The 12-day teacher training is held at each of the four districts at either NCHD office or other community space. Each training group has a maximum of 15 CYL participants. These trainings are eight hours in duration each day.  The first 10 days include icebreakers, discussion, and role-play exercises. The remaining two days are scheduled for “hands-on” practice or practicum opportunities. | During the first 10 days, CYLs will review early childhood development modules and LEAPS preschool curriculum in detail. Trainers follow a structured training manual.  During the remaining two days, CYLs will be offered opportunities to practice activities from the preschool curriculum. These practicums are held at an already running LEAPS preschool, with support from NCHD Field Officers and LIST members. | - **Community-Based Preschool Activity guide**: Details classroom-based activities which are categorized according to 50 Key Development indicators (KDIs) across the seven LEAPS learning areas. These seven learning areas include: (i) Approaches to learning; (ii) Social-emotional, and moral development; (iii) Physical development and health; (iv) Language, literacy, and communications; (v) Mathematics; and (vi) My world. - **Attendance and Enrolment Registers**: A session focusing on filling attendance and other contact information is conducted to familiarize CYLs with how to use their registers. - **Child Progress Forms**: A copy of child progress forms will be provided to CYLs. CYLs will use this form to assess children’s performance and progress toward achieving the KDIs throughout the academic year. |

**Table 5.** LEAPS Preschool Routine. Table 5 describes the LEAPS preschool routine and activities; CYL = Community Youth Leader.

| **Time** | **Session** | **Routine** |
| --- | --- | --- |
| 8:00- 8:30 am | Arrival Time and Free Play | - Meet and greet children and parents - Help children wash hands - Children self-register attendance on the attendance chart - Free Play: Children can play in groups or individually in the quiet corner designated in each classroom |
| 8:30- 8:45 am | Snack Time | - Wash hands - Children take their snack - Children clean up after snack with support from CYL |
| 8:45- 9:10 am | Circle Time | - CYLs share stories, songs, rhymes, etc. during circle time. Additionally, they use circle time to talk about the day of the week, weather, and any other special information (e.g., a child’s birthday) |
| 9:10- 9:20 am | Break | - 10-minute stretch break |
| 9:20-10:05am | Learning centers | - Individual and small group activities |
| 10:05-10:10am | Clean-up Time | - CYL sings clean up songs with children and supports them to clean up |
| 10:10-10:50 am | Large group activities | - Children play indoors or outdoors in large group (e.g., action songs or playing with a ball). |
| 10:50-11:00 am | Departure Time | - CYL reviews the day with children - Helps children to wash hands and say goodbye |

**Table 6.** LEAPS Actions for Emergency Response: Table 6 shows three response actions for LEAPS that will be implemented during times of emergencies or conflict in which school routines are disrupted. CYL= Community Youth Leader; KDIs= Key Development Indicators.

| **Response Action** | **Descriptions** |
| --- | --- |
| Remote Learning Activities for CYLs | CYLs may engage in remote learning activities ranging from writing a story to creating learning materials for their classroom. Remote-learning activities are intended to help CYLs to stay connected to LEAPS and support skill development. |
| Workbooks for Children | In order to promote learning at home and to stay connected to LEAPS, children will be given a workbook during times of emergencies or conflict. The children’s workbook will have several learning worksheets which children can complete with support from their family members and siblings. Example worksheet topics will include alphabets, numbers, transportation, local animals, fruits, and vegetables. These worksheets will be aligned with the 50 KDIs across seven learning areas. |
| Additional Needs-Based Trainings | Additional trainings will be conducted prior to school re-opening especially if schools have been shut down for a longer period of time. These trainings will help CYLs review concepts from the initial 12-day training and also address additional content arising from emergency and conflict situations which may not have been previously addressed. Where needed, additional resources such as handouts, storybooks, classroom materials, etc., will be provided to support CYLs and children. |
