# Supplementary material for: Evaluating implementation of LEAPS, a youth-led early childhood care and education intervention in rural Pakistan: protocol for a stepped wedge cluster-randomized trial
Source: Trials. 2021 Aug 17;22:542. doi: 10.1186/s13063-021-05518-9 (PMC8371849; doi:10.1186/s13063-021-05518-9)
Supplement: Supplementary file 2 — Additional file 2. Trial protocol registration and sponsor details. [file 13063_2021_5518_MOESM2_ESM.docx]

**Additional file 2. Trial protocol registration and sponsor details**

**Section 1. Protocol Version**

**Issue Date:** July 31, 2020

**Protocol Amendment Number:** 03

**Authors:** AKY, SB

**Table 1.** Revision Chronology

| **December 5, 2018** | Original |
| --- | --- |
| **January 25, 2019** | Amendment 01: Primary reason for amendment:  Updated Study status to ‘recruiting’. |
| **June 6, 2019** | Amendment 02: Primary reason for amendment:  Updates to study contacts |
| **July 31, 2020** | Amendment 03: Primary reason for amendment:  Extension of study period due to COVID-19 interruptions. Updates to IDELA scoring information. |

**Section 2. Sponsor Contact Information**

**Trial Sponsor:** Harvard

**Sponsor’s Reference:**

***Contact Name:*** Aisha K. Yousafzai

***Address:*** 665 Huntington Ave., Boston, MA 02115

***Telephone***: +1-857-318-8691

***Email***: ayousafzai@hsph.harvard.edu
